# The correlation between epicardial adipose tissue thickness and left ventricular systolic function in patients with systemic lupus erythematosus

**DOI:** 10.3389/fcvm.2026.1806431

**Published:** 2026-10-14

**Authors:** Sisi Lin, Shengmin Zhang, Hui Zhou

**Affiliations:** 1 Department of Medical Ultrasound, The Affiliated Lihuili Hospital of Ningbo University, Ningbo, China; 2 Department of Medical Ultrasound, The First Affiliated Hospital of Ningbo University, Ningbo, China

**Keywords:** epicardial adipose tissue, GLS (global longitudinal strain), left ventricular systolic function, systemic lupus erythematosus, two-dimensional speckle tracking echocardiography

## Abstract

**Background:**

Systemic lupus erythematosus (SLE) can cause myocardial damage and left ventricular dysfunction in patients. Abnormal deposition of epicardial adipose tissue (EAT) can promote the occurrence and development of cardiovascular diseases. The aim of this study was to investigate changes in EAT thickness in patients with SLE and apply two-dimensional speckle tracking echocardiography (2D-STE) to study the correlation between EAT and left ventricular systolic function.

**Patients and methods:**

This case–control study recruited a total of 67 SLE patients as the case group and 31 healthy individuals as the control group. Left ventricular systolic function parameters were measured using 2D-STE. Changes and correlations in laboratory indicators, EAT, and left ventricular systolic function parameters were compared between groups.

**Results:**

Compared with the control group, the case group showed significantly increased EAT and diastolic left ventricular posterior wall thickness (LVPWD) (*P*<0.05). High-density lipoprotein cholesterol (HDL-C), apical two-chamber longitudinal strain (AP2C LS), apical three-chamber longitudinal strain (AP3C LS), apical four-chamber longitudinal strain (AP4C LS), and global longitudinal strain (GLS) were significantly reduced (*P* < 0.05). The 18-segment left ventricular longitudinal strain was further grouped according to coronary artery supply; the LAD, RCA, and LCX groups in the patient cohort all showed reductions compared with the healthy control group (*P* < 0.05). Spearman correlation analysis revealed that EAT was negatively correlated with GLS (*r* = −0.39, *P* < 0.05), LAD group (*r* = −0.26, *P* < 0.05), RCA group (*r* = −0.47, *P* < 0.05), and LCX group (*r* = −0.29, *P* < 0.05), and positively correlated with duration of illness (DT) (*r* = 0.41, *P* < 0.05) and age (*r* = 0.45, *P* < 0.05). Multiple linear regression analysis confirmed that EAT is an important influencing factor of left ventricular longitudinal strain function (*P* < 0.05).

**Conclusion:**

Increased epicardial adipose tissue thickness is negatively correlated with left ventricular systolic function. EAT can effectively reflect changes in left ventricular systolic function in SLE patients, providing a basis for clinical management and early intervention.

## Introduction

Systemic lupus erythematosus (SLE) is a chronic systemic autoimmune disease that can severely damage organs through persistent inflammation and abnormal immune responses (1). Cardiovascular disease (CVD) is one of the leading causes of organ damage and death in these patients (2, 3). Early detection and accurate diagnosis of myocardial damage and cardiac dysfunction in SLE patients are of great significance. Epicardial adipose tissue (EAT) is a unique fat depot located between the myocardium and the visceral pericardium, unevenly surrounding the myocardial tissue and in direct contact with the myocardium and coronary arteries. Abnormal deposition of EAT can promote the occurrence and progression of CVD (4, 5). Under normal physiological conditions, EAT exerts a protective effect, providing energy to the myocardium while also secreting anti-inflammatory and anti-fibrotic adipokines. However, under pathological conditions, EAT releases pro-inflammatory mediators and exacerbates oxidative stress, thereby promoting the occurrence of CVD (6, 7). Echocardiography is a standard clinical diagnostic tool for evaluating heart structure and function (8). At present, left ventricular ejection fraction (LVEF) is the established core parameter for echocardiographic assessment of left ventricular systolic function and has been incorporated into multiple clinical guidelines and enrollment criteria for heart failure trials (9). However, LVEF is limited by load dependence and geometric assumptions, making it less sensitive to the complex mechanical changes in the myocardium. Two-dimensional STE is based on tracking the spatial motion of myocardial echo speckles and quantifies the real-time motion and deformation of myocardial tissue by analyzing their displacement trajectories (10). In particular, GLS obtained using speckle-tracking technology is superior to LVEF in assessing left ventricular systolic function, exhibiting higher reproducibility, and enabling early detection of subclinical myocardial dysfunction (11). The purpose of this study was to evaluate left ventricular systolic function in patients with SLE using 2D-STE—with a comparative analysis against healthy individuals—and to further explore the correlations between EAT and both biochemical markers and echocardiographic parameters of left ventricular systolic function in SLE patients.

## Materials and methods

### Study population

This was a single-center, case–control study. A total of 67 SLE patients admitted to our hospital from February 2025 to November 2025 were selected as the case group, and 31 healthy individuals matched for gender and age during the same period were selected as the control group. Within the case group, we further employed a cross-sectional analysis method to explore the relationship between EAT thickness and left ventricular systolic function. The inclusion criteria were based on the 2019 SLE classification criteria revised jointly by the European League Against Rheumatism (EULAR) and the American College of Rheumatology (ACR). Patients were required to first test positive for antinuclear antibodies (ANA) by immunofluorescence (titer ≥1:80) and then achieve a cumulative score of ≥10 points across seven clinical domains and three immunologic domains. The main reasons for patient admission included SLE disease activity assessment or treatment of SLE-related clinical manifestations (such as rash, arthritis, hematological involvement, and lupus nephritis). Exclusion criteria included concomitant autoimmune disorders, such as Hashimoto's thyroiditis, ankylosing spondylitis, type 1 diabetes, and multiple sclerosis; history of myocarditis, pericarditis, pericardial effusion, coronary artery disease, or direct hospital admission due to an acute cardiovascular event; preexisting valvular heart disease that may affect circulatory function or increase study-related risks; congenital heart disease; uncontrolled severe hypertension; severe arrhythmias; severe hepatic or renal insufficiency; or poor-quality echocardiographic images. This study was approved by the Affiliated Lihuili Hospital of Ningbo University (Approval No.: KY2024SL525-01). All participants provided informed consent.

### Clinical data and laboratory measurements

Demographic and anthropometric data, including age, gender, height, weight, body mass index (BMI), and body surface area (BSA), were collected from all participants.

Laboratory parameters, including triglyceride (TG), high-density lipoprotein cholesterol (HDL-C), low-density lipoprotein cholesterol (LDL-C), C-reactive protein (CRP), erythrocyte sedimentation rate (ESR), and creatine kinase-MB (CK-MB), were measured using standard biochemical methods.

In addition, medical history data were obtained specifically from SLE patients at the time of hospital admission. The presence of chest pain (CP) and chest tightness (CT) symptoms was documented based on patient-reported history; these symptoms were recorded as clinical features for analysis rather than as the primary reasons for admission. Glucocorticoid (GC) therapy use was also recorded for the SLE case group.

### Echocardiographic data measurement

Ultrasound examinations were performed using a Philips EPIQ CVx color Doppler ultrasound diagnostic system (Philips Ultrasound, Inc., Bothell, Washington, USA) equipped with an S5-1 probe. All subjects were examined in the left lateral decubitus position and with synchronized electrocardiogram monitoring. All measurements and recordings were performed by the same experienced sonographer, with data analysis conducted using QLAB 15.0 workstation software. The left ventricular end-diastolic diameter (LVEDD), left ventricular end-systolic diameter (LVESD), interventricular septal thickness (IVSD), and left ventricular posterior wall thickness (LVPWD) were measured using two-dimensional echocardiography in the parasternal long-axis view of the left ventricle. Pulsed Doppler measurement was utilized to determine the peak early diastolic blood flow velocity (E) and peak late diastolic blood flow velocity (A) at the mitral valve orifice; the E/A ratio was calculated. Tissue Doppler measurement was applied to calculate the average peak velocity of the left ventricular lateral wall and interventricular septum at the mitral valve annulus during early diastole (e'), and the E/e' value was calculated.

The end-systolic EAT thickness was measured in various planes using echocardiography. Measurements were taken perpendicular to the right ventricular wall in the parasternal long-axis view (PLAX) (Figure 1A), parasternal short-axis view at the papillary muscles (PMPSAX), and parasternal four-chamber view (P4C) (Figure 1B). Measurements were taken perpendicular to the apex in the apical four-chamber view (A4C). Measurements were taken continuously over three cardiac cycles, and the average value as recorded.

**Figure 1:**
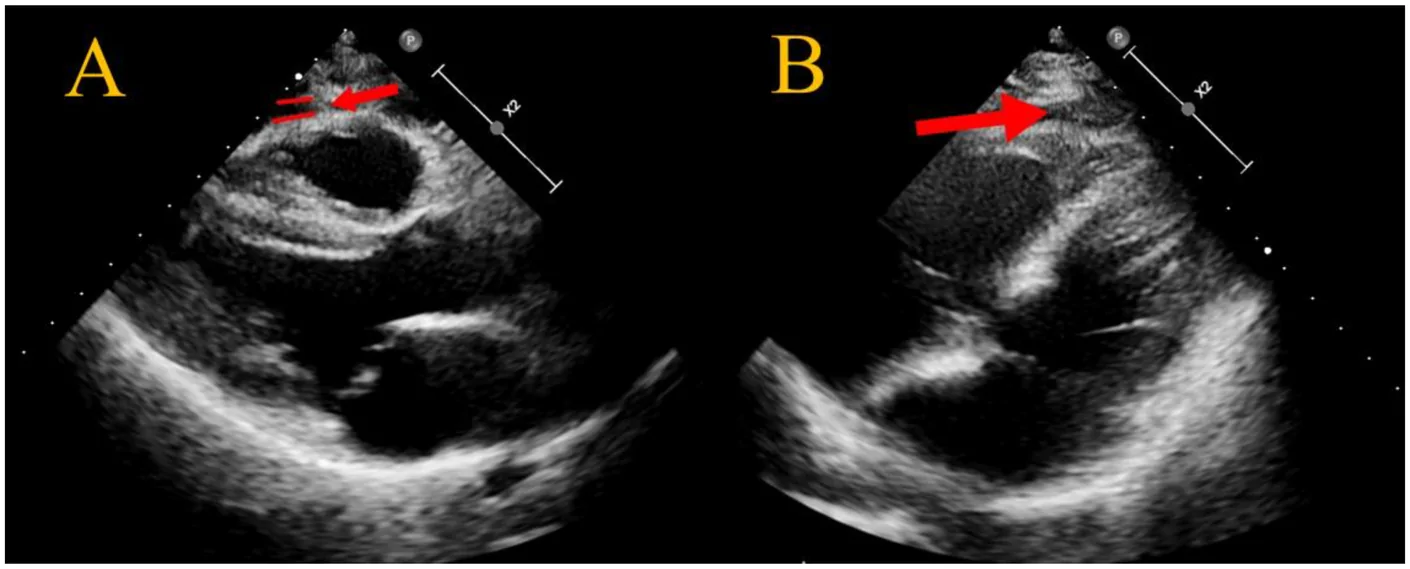
EAT thickness on the parasternal long-axis view (**A**) and the parasternal four-chamber view (**B**) of echocardiography.

Dynamic two-dimensional images were acquired and stored from apical two-chamber, three-chamber, and four-chamber heart views. A high frame rate (＞70 frames/s) was employed to accurately capture the rapid movement of the left ventricular wall myocardium during the cardiac cycle. The end of the T-wave on the electrocardiogram was used as a reference point for left ventricular end-systole. Using the aCMQ feature in QLAB software, a region of interest (ROI) encompassing the entire myocardial thickness was generated. The operator manually adjusted the width of the ROI. Automatic measurement was performed for left ventricular myocardial longitudinal strain, including AP2C LS, AP3C LS, AP4C LS, GLS (derived from the 18-segment left ventricular model), and left ventricular ejection fraction (LVEF) (Figure 2). The 18-segment longitudinal strain of the left ventricular wall was divided into three corresponding groups according to the typical distributions of the right coronary artery (RCA), left anterior descending artery (LAD), and left circumflex artery (LCX).

**Figure 2:**
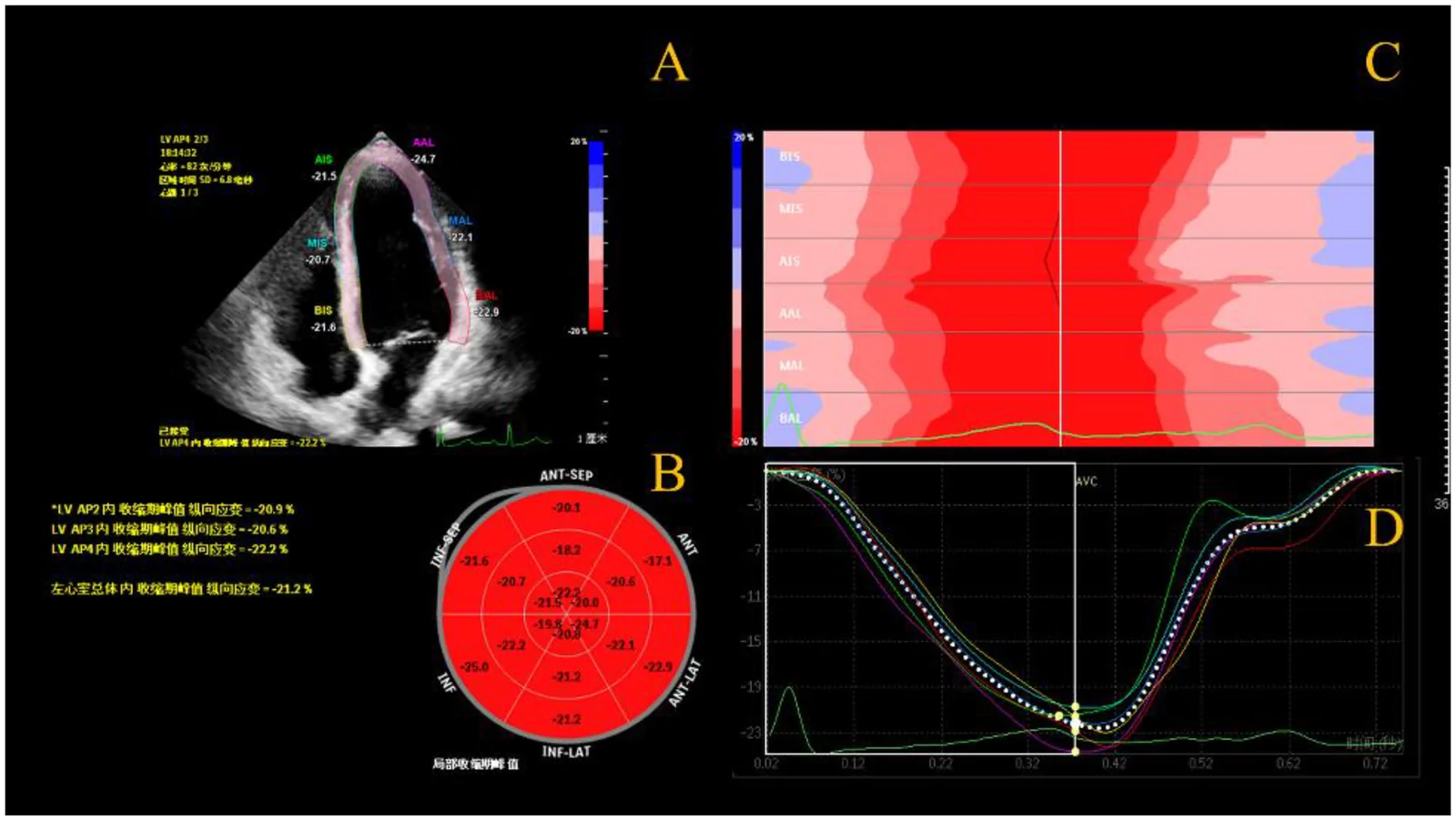
Two-dimensional speckle tracking echocardiography of the left ventricular apical four-chamber view **(A)**, left ventricular longitudinal strain bull's eye plot **(B),** M-mode strain imaging of the left ventricular four-chamber view **(C)**, left ventricular four-chamber strain-time curve **(D).**

## Statistical analysis

All statistical analyses were performed using SPSS version 27.0. Continuous variables were first tested for normality using the Kolmogorov–Smirnov test. Variables with a normal distribution were expressed as mean ± standard deviation (SD), and comparisons between two groups were performed using independent-sample t-tests. Non-normally distributed continuous variables were expressed as median with interquartile range M (P25, P75), and intergroup comparisons were conducted using the Mann–Whitney *U* test.

Spearman's rank correlation analysis was performed to evaluate the univariate associations of EAT with various clinical, laboratory, and echocardiographic parameters. In addition, univariate associations between GLS and these parameters were also examined using the same method.

To identify independent determinants of left ventricular systolic function, multivariate linear regression analysis was performed with GLS as the dependent variable. Candidate independent variables entered into the model included EAT, sex, BMI, disease duration, and all parameters that showed a significant correlation with GLS (*P* < 0.05) in the univariate analysis. Multicollinearity was first assessed using the variance inflation factor (VIF); variables with a VIF > 5 were excluded. Subsequently, a backward elimination procedure was applied to select the final set of independent predictors. A two-sided *P*-value < 0.05 was considered statistically significant.

## Results

### Comparison of clinical data and laboratory test results

Compared to the control group, the HDL-C level in the case group was reduced (*P* < 0.05). There were no statistically significant differences in clinical data such as age, gender, height, weight, BMI, body surface area, TG, and LDL-C between the two groups (*P* > 0.05) (Table 1).

**Table 1:** Comparison of clinical data and laboratory test results.

Descriptor	The control group(*n* = 31)	The case group(*n* = 67)	*p*
Age (years)	43 (35,57)	42 (33,57)	0.945
Female *n* (%)	27 (87.10%)	62 (92.54%)	0.388
Height (cm)	160 (158,166)	160 (158,165)	0.762
Weight (kg)	58 (54,65)	56 (50,62)	0.296
BMI (kg/m^2^)	22.74 ± 3.42	22.10 ± 3.39	0.386
BSA (m^2^)	1.62 (1.51,1.72)	1.57 (1.50,1.66)	0.232
TG (mmol/L)	1.18 (0.84,1.70)	1.38 (0.92,1.99)	0.246
HDL-C (mmol/L)	1.61 ± 0.34	1.38 ± 0.38	0.007**
LDL-C (mmol/L)	2.57 (2.10,2.95)	2.45 (1.95,2.91)	0.253

BMI, body mass index; BSA, body surface area; TG, triglycerides; HDL-C, high-density lipoprotein cholesterol; LDL-C, low-density lipoprotein cholesterol.

**p < 0.01.

### Comparison of conventional echocardiography parameters and EAT data

Compared with the control group, LVPWD thickened in the case group (*P* < 0.05). There were no significant differences in cardiac structural indexes and diastolic functional parameters, including LVEDD, LVESD, IVSD, E/A, and E/e', between the two groups (*P* > 0.05). Compared with the control group, PLAX EAT, PMPSAX EAT, P4C EAT, A4C EAT, and mean EAT in the case group were all thickened, and the differences were statistically significant (*P* < 0.05) (Table 2; Figure 3).

**Table 2:** Comparison of conventional echocardiography parameters and EAT data.

Parameter	The control group (*n* = 31)	The case group (*n* = 67)	*p*
LVEDD (mm)	46 (44,49)	45 (42,48)	0.093
LVESD (mm)	30 (27,32)	29 (26,32)	0.328
IVSD (mm)	9.8 (8.8,11.1)	10.2 (8.8,11.4)	0.677
LVPWD (mm)	8.6 (8.2,9.8)	9.4 (8.6,10.7)	0.046*
E/A	1.1 (0.8,1.6)	1.2 (0.8,1.7)	0.574
E/e’	7.2 (5.8,8.2)	7.3 (6.1,8.2)	0.912
Mean EAT (mm)	4.1 ± 1.0	5.0 ± 1.2	0.001**
PLAX EAT (mm)	4.0 ± 1.3	4.8 ± 1.4	0.010*
PMPSAX EAT (mm)	4.3 ± 1.1	4.9 ± 1.3	0.032*
P4C EAT (mm)	4.4 ± 1.1	5.1 ± 1.4	0.013*
A4C EAT (mm)	3.9 ± 1.1	5.0 ± 1.5	0.000**

LVEDD, left ventricular end-diastolic diameter; LVESD, left ventricular end-systolic diameter; IVSD, interventricular septal thickness; LVPWD, left ventricular posterior wall thickness; E/A, the E/A ratio; E, peak early diastolic blood flow velocity; A, peak late diastolic blood flow velocity; E/e', the E/e' value; e', the average peak velocity of the left ventricular lateral wall and interventricular septum at the mitral valve annulus during early diastole; Mean EAT, four average EAT thickness of the measurement planes; PLAX EAT, EAT thickness of the parasternal long-axis view; PMPSAX EAT, EAT thickness on the parasternal short-axis view of the papillary muscles; P4C EAT, EAT thickness of the parasternal four-chamber view; A4C EAT, EAT thickness of the apical four-chamber view.

*p < 0.05.

**p < 0.01.

**Figure 3:**
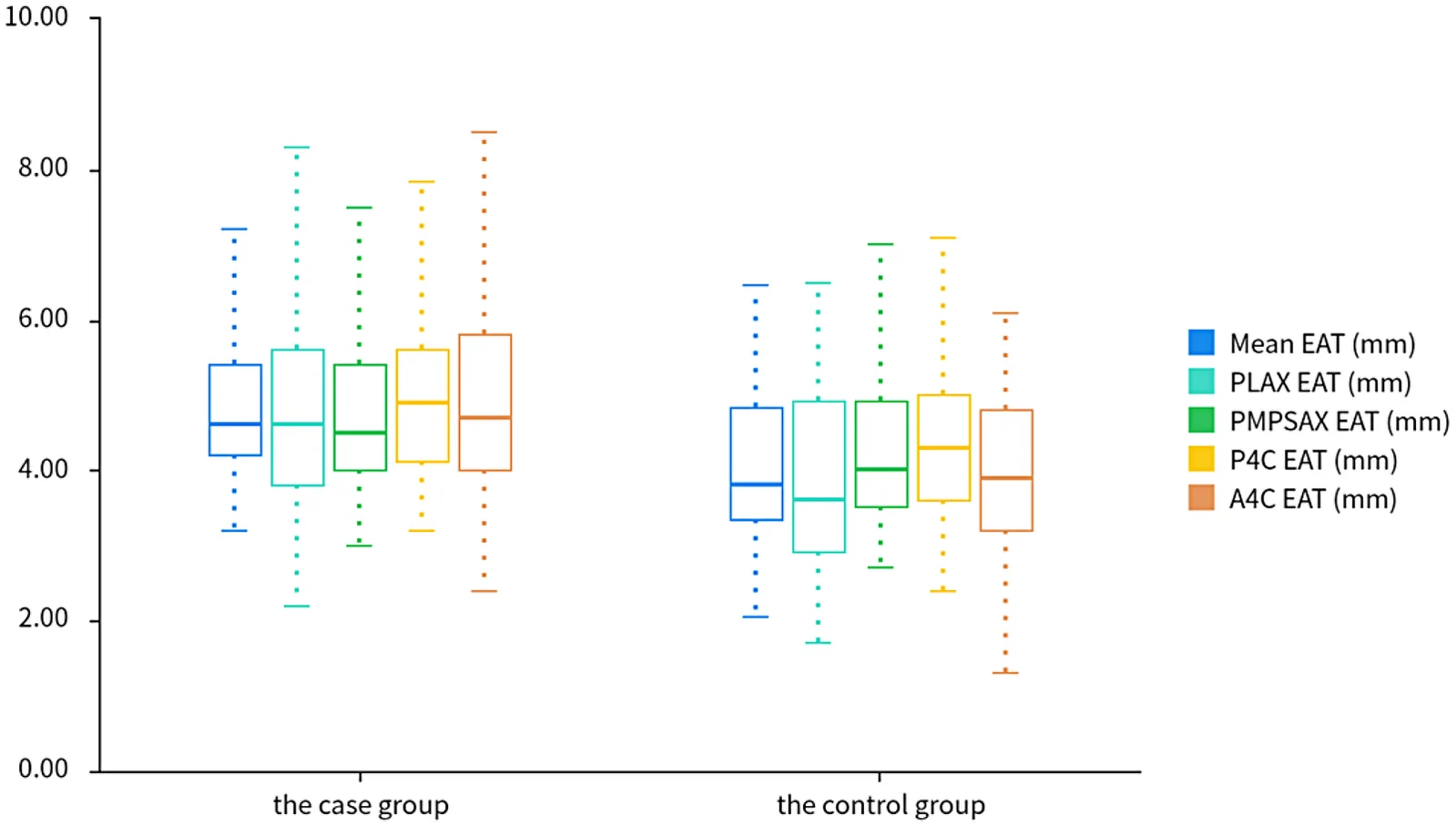
Specific differences in EAT thickness between the control group and the case group; compared with the control group, the PLAX EAT, PMPSAX EAT, P4C EAT, A4C EAT, and mean EAT in the case group were all thicker, and the differences were statistically significant.

### Comparison of longitudinal strain parameters of left ventricular myocardium

Compared with the control group, the AP2C LS, AP3C LS, AP4C LS, and GLS in the case group were all reduced (*P* < 0.05); there was no significant difference in LVEF between the two groups (*P* > 0.05) (Table 3).

**Table 3:** Comparison of longitudinal strain parameters of left ventricular myocardium.

Parameter	The control group (*n*=31)	The case group (*n*=67)	*p*
AP2C LS (%)	−19.9 ± 2.6	−18.4 ± 2.5	0.007**
AP3C LS (%)	−20.1 ± 3.0	−17.6 ± 3.0	0.000**
AP4C LS (%)	−20.0 ± 2.3	−18.6 ± 3.1	0.030*
GLS (%)	−20.0 ± 2.0	−18.2 ± 2.2	0.000**
LVEF (%)	59.24 ± 6.72	56.95 ± 6.44	0.110

AP2C LS, apical two-chamber longitudinal strain; AP3C LS, apical three-chamber longitudinal strain; AP4C LS, apical four-chamber longitudinal strain; GLS, Global longitudinal strain; LVEF, left ventricular ejection fraction.

*p < 0.05.

**p < 0.01.

### 18-segment left ventricular longitudinal strain parameters and group comparison

Compared with the control group, AL-B, IS-B, AS-B, AL-M, INF-M, IS-M, and AL-A decreased in the case group (*P* < 0.05) (Table 4). The 18-segment longitudinal strain of the left ventricular wall was grouped according to the typical distribution of the right coronary artery (RCA), left anterior descending artery (LAD), and left circumflex artery (LCX) (12). Compared with the control group, the values were lower in the LAD, RCA, and LCX subgroups of the case group (Table 5; Figures 4–7).

**Table 4:** Comparison of longitudinal strain values in each segment of the left ventricular wall.

Descriptor (%)	The control group (*n* = 31)	The case group (*n* = 67)	*p*
ANT-B	−18.2(−20.0, −16.5)	−17.2(−19.5, −15.4)	0.118
AL-B	−18.5 ± 3.2	−15.9 ± 4.2	0.003**
IL-B	−15.8(−18.1, −13.8)	−14.4(−16.9, −11.9)	0.092
INF-B	−16.9 ± 4.1	−15.6 ± 4.3	0.169
IS-B	−20.1(−25.2, −15.2)	−17.3(−19.7, −13.7)	0.011**
AS-B	−19.4 ± 4.0	−17.2 ± 4.6	0.028*
ANT-M	−19.9(−22.1, −17.3)	−17.6(−21.2, −15.5)	0.067
AL-M	−20.5 ± 4.0	−18.6 ± 3.7	0.021*
IL-M	−20.2 ± 3.1	−19.1 ± 3.5	0.127
INF-M	−20.4 ± 3.0	−18.4 ± 3.4	0.006**
IS-M	−20.0 ± 3.3	−16.7 ± 3.6	0.000**
AS-M	−19.2 ± 3.1	−18.7 ± 3.8	0.482
ANT-A	−20.1 ± 4.2	−18.8 ± 5.0	0.222
AL-A	−22.6 ± 5.3	−19.5 ± 4.7	0.005**
IL-A	−23.8 ± 3.9	−22.3 ± 4.9	0.133
INF-A	−23.3 ± 4.3	−21.8 ± 4.9	0.156
IS-A	−21.3 ± 7.7	−18.8 ± 6.4	0.098
AS-A	−21.0 ± 5.1	−19.8 ± 5.5	0.310

Ant-b, basal anterior; Al-B, basal anteroseptal; Il-B, basal inferoseptal; Inf-B, basal inferior; Is-B, basal inferolateral; As-B, basal anterolateral; Ant-M, mid anterior; Al-M, mid anteroseptal; Il-M, mid inferoseptal; Inf-M, mid inferior; Is-M, mid inferolateral; As-M, mid anterolateral; Ant-A, apical anterior; Al-A, apical anteroseptal; Il-A, apical inferoseptal; Inf-A, apical inferior; Is-A, apical inferolateral; As-A, apical anterolateral.

*p < 0.05.

**p < 0.01.

**Table 5:** Comparison of longitudinal strain in left ventricular wall segments grouped by coronary artery supply.

Descriptor (%)	The control group (*n* = 31)	The case group (*n* = 67)	*p*
LAD Group	−20.9 ± 2.7	−19.0 ± 2.8	0.002**
RCA Group	−18.4 ± 2.7	−16.8 ± 3.1	0.020*
LCX Group	−19.7 ± 2.5	−17.4 ± 2.9	0.000**

LAD, left anterior descending artery; RCA, right coronary artery; LCX, left circumflex artery.

*p < 0.05.

**p < 0.01.

**Figure 4:**
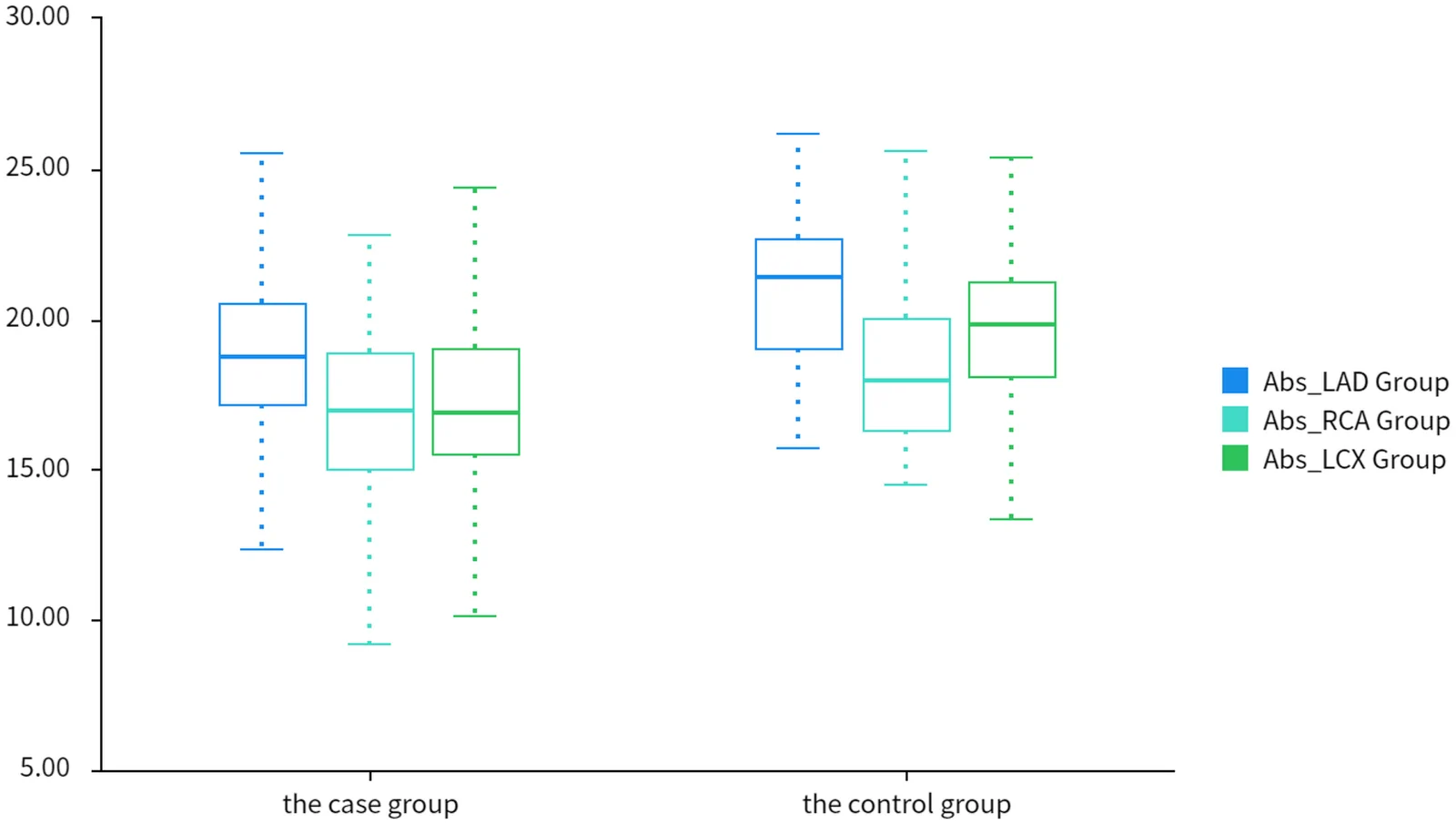
The specific differences in longitudinal strain values of left ventricular wall segments grouped by coronary arteries between the control group and the case group. Abs-LAD group, absolute value of LAD group; Abs-RCA group, absolute value of RCA group; Abs-LCX group, absolute value of LCX group; the parameter level for each group is determined based on the absolute value. Compared with the control group, the case group showed decreased levels in Abs-LAD group, Abs-RCA group, and Abs-LCX group, and the differences were statistically significant.

**Figure 5:**
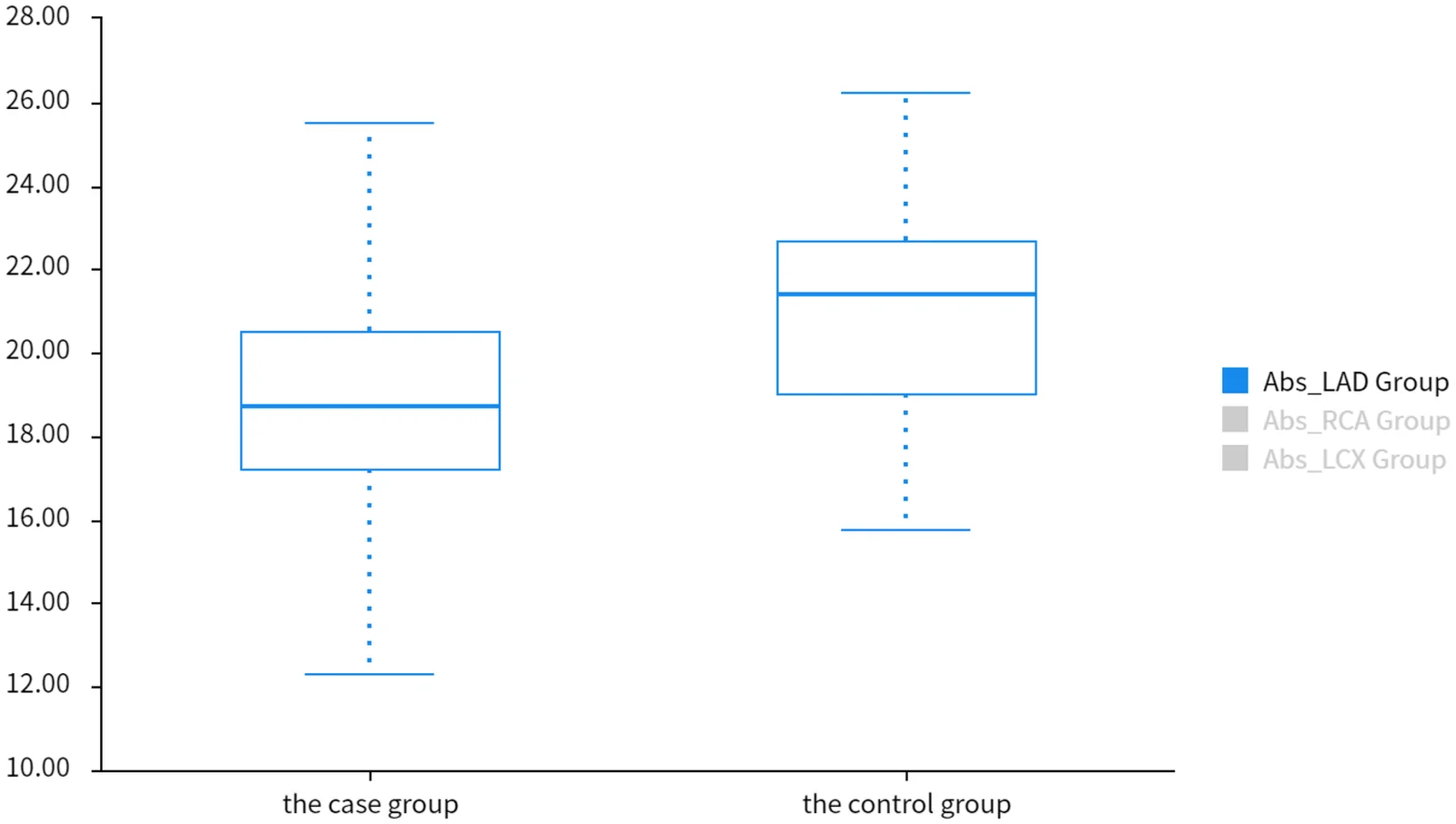
Specific differences in Abs-LAD group between the control group and the case group. Abs-LAD group, absolute value of LAD group; the parameter level for each group is determined based on the absolute value. Compared with the control group, the case group showed decreased levels in Abs-LAD group, and the differences were statistically significant.

**Figure 6:**
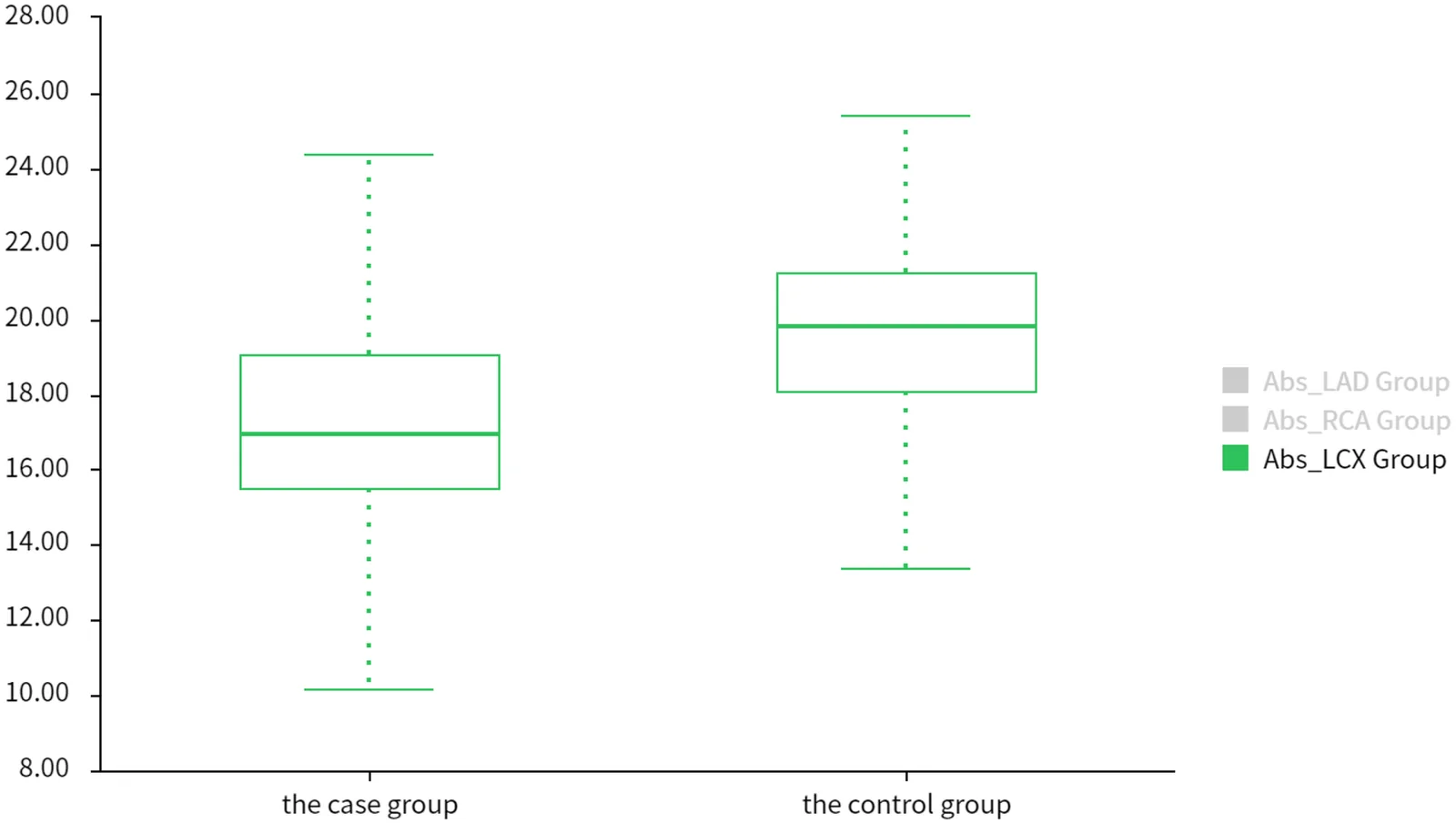
Specific differences in Abs-LCX group between the control group and the case group. Abs-LCX group, absolute value of LCX group; the parameter level for each group is determined based on the absolute value. Compared with the control group, the case group showed decreased levels in Abs-LCX group, and the differences were statistically significant.

**Figure 7:**
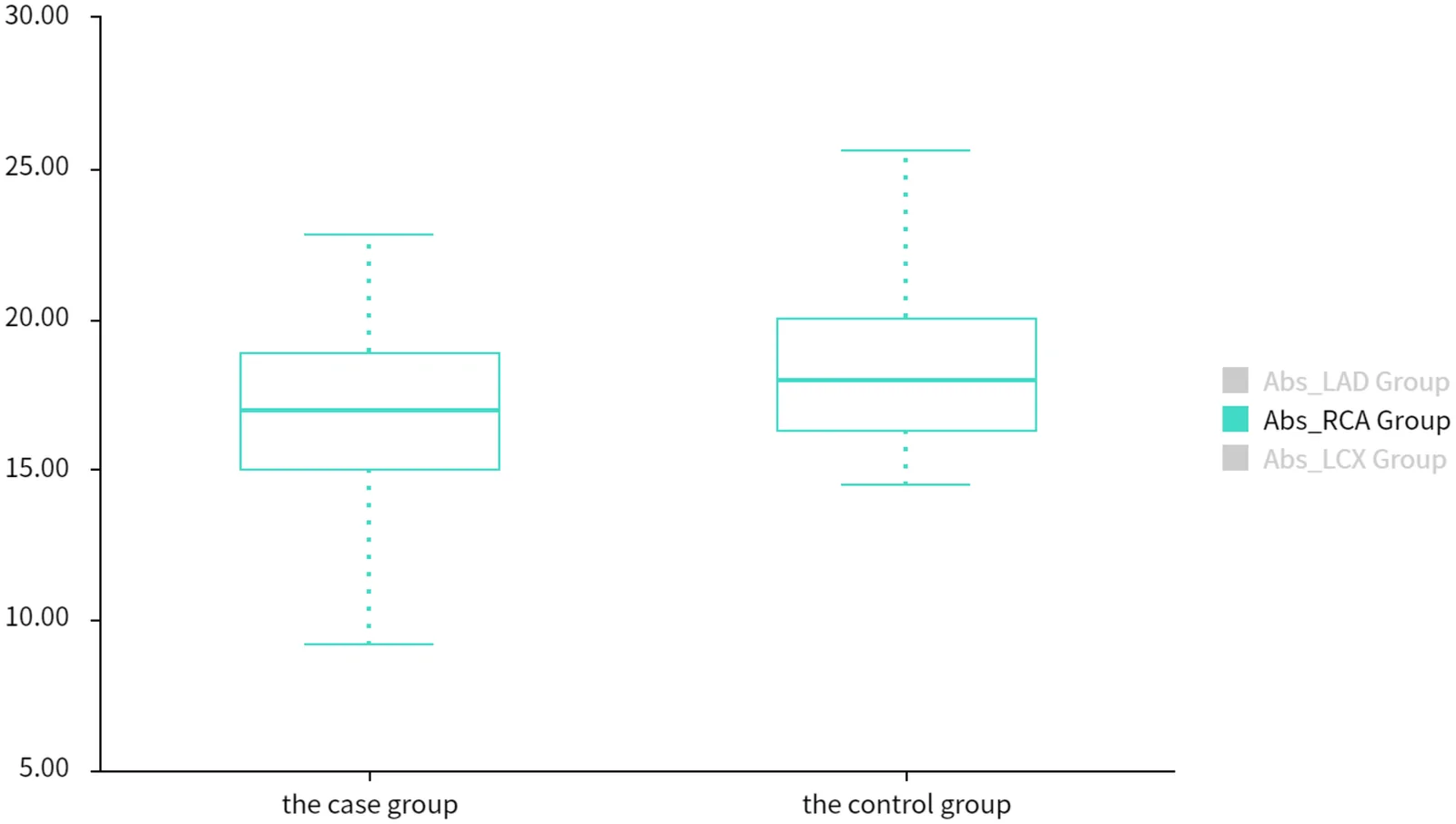
Specific differences in Abs-RCA group between the control group and the case group. Abs-RCA group, absolute value of RCA group; the parameter level for each group is determined based on the absolute value. Compared with the control group, the case group showed decreased levels in Abs-RCA group, and the differences were statistically significant.

**Figure 8:**
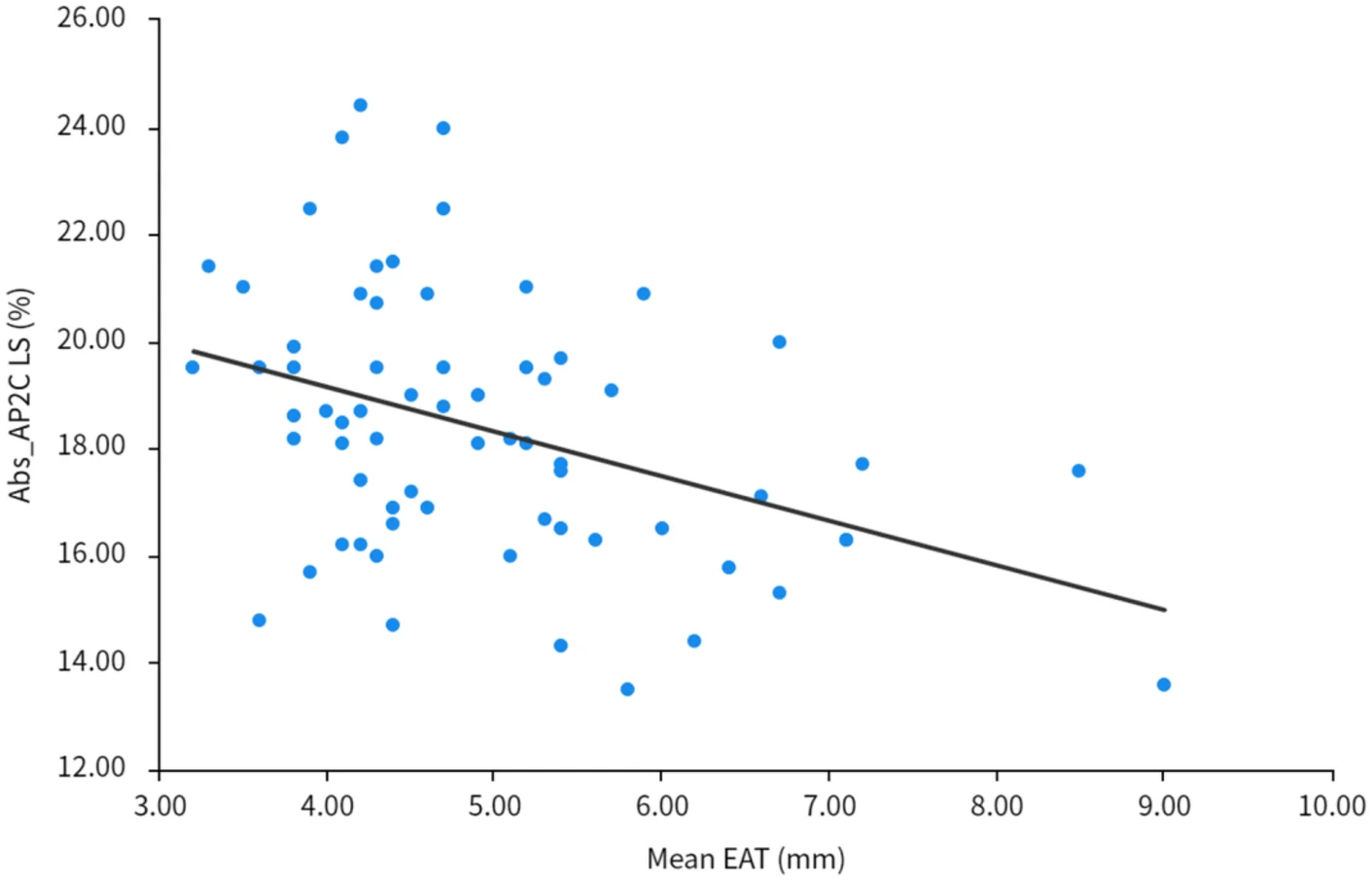
Correlation between mean EAT and Abs-AP2C LS. Abs-AP2C LS, absolute value of AP2C LS; AP2C LS determines parameter levels based on the absolute value size. Mean EAT and AP2C LS are negatively correlated.

**Figure 9:**
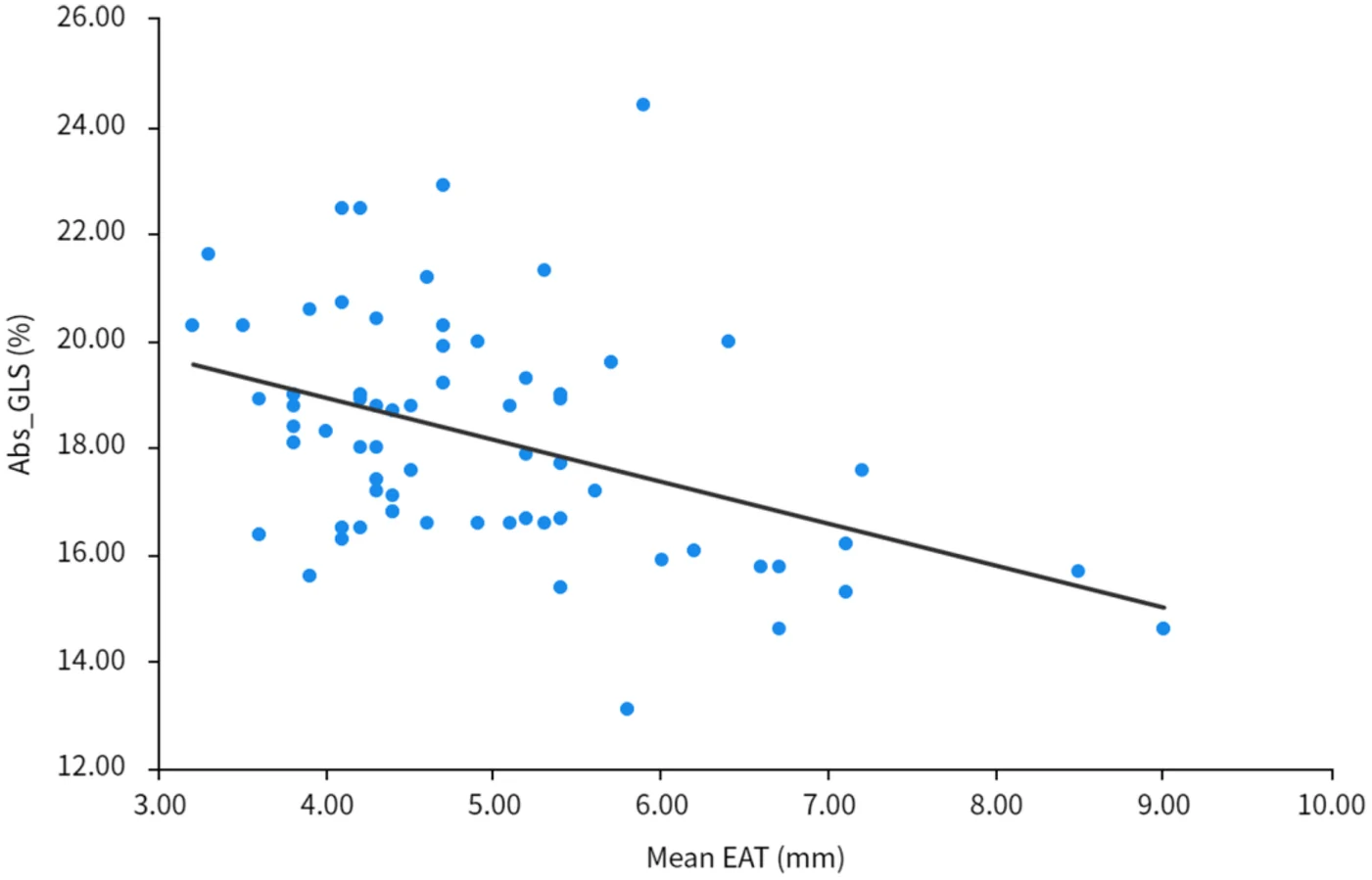
Correlation between mean EAT and Abs-GLS. Abs-GLS, absolute value of GLS; GLS determines parameter levels based on the absolute value size. Mean EAT and GLS are negatively correlated.

### Correlation analysis

Spearman correlation analysis revealed that various EAT measurements were predominantly negatively correlated with left ventricular global and segmental longitudinal strain parameters, including AP2C LS, AP3C LS, AP4C LS, and GLS, with the most consistent and strongest correlations observed for GLS and AP2C LS (Figures 8, 9). With regard to coronary artery supply territories, EAT parameters showed the strongest negative correlations with the RCA territory, followed by the LCX territory, while correlations with the LAD territory were relatively weaker. In addition, most EAT measurements were significantly correlated with diastolic function parameters (E/A and E/e'), left ventricular hypertrophy indices (IVSD and LVPWD), DT, and age, suggesting that EAT is closely associated with impaired systolic and diastolic functions, myocardial remodeling, and advancing age. However, no significant correlations were found between EAT and conventional left ventricular ejection fraction (LVEF) or most laboratory parameters, including inflammatory markers. Detailed correlation coefficients and corresponding *P* values for all variables are presented in Table 6.

**Table 6:** Correlation analysis.

Parameter	Correlation coefficient				
	Mean EAT	PLAX EAT	PMPSAX EAT	P4C EAT	A4C EAT
AP2C LS	−0.363**	−0.245*	−0.297*	−0.386**	−0.403**
AP3C LS	−0.254*	−0.251*	−0.276*	−0.197	−0.194
AP4C LS	−0.258*	−0.188	−0.287*	−0.128	−0.344**
GLS	−0.388**	−0.318**	−0.379**	−0.315**	−0.411**
LVEF	0.068	0.048	−0.050	0.041	0.065
LAD group	−0.262*	−0.128	−0.206	−0.264*	−0.260*
RCA group	−0.469**	−0.450**	−0.451**	−0.324**	−0.466**
LCX group	−0.288*	−0.337**	−0.313**	−0.135	−0.319**
DT	0.413**	0.419**	0.287*	0.297*	0.414**
Age	0.452**	0.431**	0.440**	0.469**	0.243*
Female	−0.026	0.001	0.055	−0.112	−0.040
BMI	0.180	0.135	0.266*	0.190	0.062
BSA	−0.005	−0.001	0.121	−0.066	−0.062
TG	0.167	0.084	0.300*	0.187	0.040
LDL-C	−0.039	−0.094	−0.040	−0.020	0.029
HDL-C	−0.042	−0.126	−0.048	−0.037	0.075
CRP	−0.039	−0.008	0.035	0.036	−0.072
ESR	−0.026	0.021	−0.078	−0.003	−0.048
CK-MB	−0.108	−0.005	−0.156	−0.037	−0.123
CP	−0.013	−0.102	0.038	−0.038	0.006
CT	−0.154	−0.201	−0.067	−0.095	−0.060
GC	0.093	0.140	0.082	0.020	−0.031
HLN	0.088	0.071	0.084	0.038	0.138
LVEDD	0.187	0.161	0.258*	0.201	0.041
LVESD	0.167	0.147	0.179	0.056	0.126
IVSD	0.375**	0.347**	0.368**	0.333**	0.353**
LVPWD	0.463**	0.427**	0.444**	0.378**	0.363**
E/A	−0.304*	−0.238	−0.318**	−0.267*	−0.249*
E/e’	0.374**	0.302*	0.431**	0.462**	0.121

Mean EAT, four average EAT thickness of the measurement planes; PLAX EAT, EAT thickness of the parasternal long-axis view; PMPSAX EAT, EAT thickness on the parasternal short-axis view of the papillary muscles; P4C EAT, EAT thickness of the parasternal four-chamber view; A4C EAT, EAT thickness of the apical four-chamber view; AP2C LS, apical two-chamber longitudinal strain; AP3C LS, apical three-chamber longitudinal strain; AP4C LS, apical four-chamber longitudinal strain; GLS, global longitudinal strain; LVEF, left ventricular ejection fraction; LAD, left anterior descending artery; RCA, right coronary artery; LCX, left circumflex artery; DT, duration of illness; BMI, body mass index; BSA, body surface area; TG, triglycerides; HDL-C, high-density lipoprotein cholesterol; LDL-C, low-density lipoprotein cholesterol; CRP, C-reactive protein; ESR, erythrocyte sedimentation rate; CK-MB, creatine kinase-MB; CP, chest pain; CT, chest tightness; GC, glucocorticoid; HLN, history of lupus nephritis; LVEDD, left ventricular end-diastolic diameter; LVESD, left ventricular end-systolic diameter; IVSD, interventricular septal thickness; LVPWD, left ventricular posterior wall thickness; E/A, the E/A ratio; E, peak early diastolic blood flow velocity; A, peak late diastolic blood flow velocity; E/e', the E/e' value; e', the average peak velocity of the left ventricular lateral wall and interventricular septum at the mitral valve annulus during early diastole.

*p < 0.05.

**p < 0.01.

### Multiple linear regression analysis

To identify independent determinants of GLS, we performed a multiple linear regression analysis. GLS was entered as the dependent variable, while mean EAT, DT, sex, BMI, LDL-C, HDL-C, and the E/A ratio were included as independent variables.

Among the included variables, mean EAT emerged as the strongest and only statistically significant independent predictor of GLS (*β* = –0.497, *p* < 0.001), with a non-standardized coefficient of −0.932. This indicates that for every 1-mm increase in mean EAT thickness, GLS decreased by approximately 0.93 percentage points, reflecting a clinically meaningful negative association after adjusting for potential confounders.

Collectively, these results demonstrate that after adjusting for traditional cardiovascular risk factors and echocardiographic parameters, mean EAT remains an important determinant of left ventricular longitudinal systolic function, as assessed by GLS. This finding underscores the potential role of EAT in subclinical myocardial dysfunction beyond conventional metabolic indicators (Table 7).

**Table 7:** Multiple linear regression analysis.

Parameter	Non-standardized coefficient		Standardization coefficient	*t*	*p*	Collinearity diagnosis	
	*B*	Standard error	*Beta*			VIF	Tolerance
Mean EAT	−0.932	0.236	−0.497	−3.94	0.000**	1.389	0.720
DT	−0.033	0.035	−0.114	−0.943	0.349	1.278	0.783
Female	−1.877	0.981	−0.226	−1.915	0.06	1.216	0.822
BMI	0.097	0.077	0.15	1.268	0.21	1.218	0.821
LDL-C	−0.522	0.285	−0.217	−1.83	0.072	1.226	0.816
HDL-C	1.423	0.717	0.247	1.985	0.052	1.351	0.74
E/A	−0.684	0.464	−0.172	−1.473	0.146	1.193	0.838
R^2^	0.324						
Adjusted R^2^	0.244						
F	F (7,59) = 4.037, *p* = 0.001						
D-W	1.858						

With GLS as the dependent variable and each research parameter as the independent variable; Mean EAT, four average EAT thickness of the measurement planes; GLS, global longitudinal strain; DT, duration of illness; BMI, body mass index; HDL-C, high-density lipoprotein cholesterol; LDL-C, low-density lipoprotein cholesterol; E/A, the E/A ratio; E, peak early diastolic blood flow velocity; A, peak late diastolic blood flow velocity.

**p < 0.01.

### Assessment of measurement reliability

To assess intraobserver and interobserver reproducibility of the measurement results, this study randomly selected 10 enrolled patients for analysis. Two experienced ultrasound physicians independently measured the images to evaluate interobserver reliability; meanwhile, one of the physicians remeasured the same set of images in a blinded state to evaluate intraobserver reliability. The intraclass correlation coefficient (ICC) was used to evaluate consistency, and its 95% confidence interval (95% CI) was calculated. The results indicate that AP2C LS, AP3C LS, AP4C LS, GLS, and Mean EAT showed good repeatability for all evaluation parameters. The intraobserver ICC values ranged from 0.859 to 0.981, and the interobserver ICC values ranged from 0.844 to 0.969 (Table 8).

**Table 8:** Assessment of measurement reliability.

Parameter	Intraobserver		Interobserver	
	ICC	95% CI	ICC	95% CI
AP2C LS	0.972	0.893–0.993	0.969	0.874–0.992
AP3C LS	0.859	0.466–0.964	0.844	0.371–0.961
AP4C LS	0.907	0.648–0.976	0.891	0.561–0.973
GLS	0.981	0.928–0.995	0.954	0.815–0.989
Mean EAT	0.941	0.775–0.985	0.860	0.436–0.965

AP2C LS, apical two-chamber longitudinal strain; AP3C LS, apical three-chamber longitudinal strain; AP4C LS, apical four-chamber longitudinal strain; GLS, global longitudinal strain; mean EAT, four average EAT thickness of the measurement planes.

## Discussion

Several core findings of this study correlate with one another, collectively suggesting a significant association between EAT thickening and early left ventricular systolic dysfunction in SLE patients. In particular, compared with the control group, the case group exhibited increased EAT thickness and significantly reduced AP2C LS, AP3C LS, AP4C LS, and GLS. Moreover, changes in left ventricular longitudinal strain function preceded alterations in conventional cardiac function parameters such as LVEF. Within the case group, EAT thickness was significantly negatively correlated with AP2C LS, AP3C LS, AP4C LS, and GLS, and EAT emerged as an important correlative factor for left ventricular longitudinal strain function. In addition, analysis based on coronary artery perfusion territories further revealed that longitudinal strain of the left ventricular segments corresponding to the LAD, RCA, and LCX territories was significantly impaired compared with healthy controls and showed a significant negative correlation with EAT thickness. Taken together, these findings indicate that EAT thickening may be closely related to early myocardial dysfunction in SLE patients, but given the observational nature of this study, causal relationships cannot be established. Despite this limitation, 2D-STE appears to provide a sensitive and feasible non-invasive assessment tool for detecting subclinical cardiac dysfunction in this population.

### Assessment of left ventricular systolic function

Two-dimensional STE is a novel ultrasound technique developed in recent years. Among its parameters, GLS has been validated by multiple studies as a sensitive indicator for assessing left ventricular systolic function; it not only enables early detection of subclinical myocardial injury in patients with normal LVEF but also possesses independent prognostic predictive value (9). In terms of ultrasound indicator selection, this study simultaneously used LVEF and GLS to evaluate left ventricular systolic function.

GLS is also superior to conventional LVEF in certain aspects. For example, GLS measurements of left ventricular function remain accurate even in the presence of mitral regurgitation (MR). Conventional LVEF often exhibits pseudo-normalization or elevation due to blood regurgitating into the left atrium in MR, thereby masking true myocardial injury. GLS directly assesses the longitudinal shortening capacity of myocardial fibers and is relatively less affected by regurgitant load, making it a superior indicator for evaluating left ventricular function in such patients.

### Cardiac function changes in SLE patients

Patients with systemic lupus erythematosus (SLE) experience accelerated atherosclerosis progression due to persistent inflammation and abnormal immune responses, which subsequently leads to the development of cardiovascular disease (CVD) (13, 14). CVD represents one of the primary causes of mortality in SLE patients (15).

In SLE patients, multiple factors including type I interferons, pro-inflammatory cytokines, inflammatory cells, immune complexes, co-stimulatory molecules, neutrophil extracellular traps, autoantibodies, oxidative stress, and dyslipidemia act together, accompanied by impaired function of endothelial progenitor cells (EPCs), leading to endothelial dysfunction and consequently a significantly increased risk of cardiovascular diseases (14). Compared to the general population, the overall risk of CVD in SLE patients is twice that of the general population (16).

Functional and structural abnormalities in the left ventricle are the most significant manifestations of inflammatory myocardial injury in SLE (17). Previous studies have confirmed that STE can be used to assess changes in left ventricular function in SLE patients (18). Endocardial myocardial tissue in SLE patients is most susceptible to specific damage, and 2D-STE endocardial myocardial strain detection can sensitively detect subclinical myocardial injury at an early stage (19). Existing studies have shown that even with normal LVEF, patients with SLE still have myocardial injury and left ventricular remodeling (20). In the study by Farag et al., it was found that patients with SLE had early involvement of GLS before the deterioration of LVEF (21). Similarly, Giollo et al. found that GLS was significantly lower in most myocardial segments in SLE patients compared with the control group (*P* < 0.05) (22). In a study by Huang et al., patients with SLE and subclinical myocardial damage had lower LVGLS relative to both SLE controls without myocardial involvement and healthy controls (*P* < 0.05). (23). These findings are consistent with those of the present study, in which AP2C LS, AP3C LS, AP4C LS, and GLS values were lower in the case group than in the control group (*P* < 0.05), whereas LVEF showed no significant intergroup differences.

At the same time, compared with the control group, the case group showed increased LVPWD and decreased HDL-C (*P* < 0.05), which may be associated with myocardial injury and CVD progression in SLE patients. Studies have shown that too high and too low HDL-C levels indicate an increased risk of all-cause mortality, with cardiovascular disease and cancer risks being particularly significant (24). In cardiovascular diseases, high-density lipoprotein (HDL) loses its normal function and instead exhibits pro-inflammatory and pro-oxidative properties. This not only prevents it from exerting its protective effects but also promotes the formation of oxidized low-density lipoprotein (OxLDL) and induces the release of chemokines and cytokines. Subsequently, macrophages take up large amounts of OxLDL through CD36 receptors on their surface, forming foam cells and ultimately promoting the development of atherosclerotic plaques (13).

### The relationship between EAT and cardiac function changes

Due to its direct anatomical adjacency to the myocardium and coronary arteries without fascial separation, EAT regulates cardiac function primarily through paracrine and vasocrine pathways, exhibiting distinct roles under physiological and pathological conditions.

Under physiological conditions, EAT exerts cardioprotective effects, including mechanical buffering against coronary torsional stress, provision of the majority of free fatty acids for myocardial ATP production (25), secretion of anti-inflammatory adipokines such as adiponectin (26, 27), and UCP-1-mediated non-shivering thermogenesis (4, 28). These actions maintain a balance between anti-inflammatory and pro-inflammatory states, thereby preserving cardiac homeostasis (4, 29).

Under pathological conditions, EAT shifts toward a detrimental phenotype, characterized by the following: (i) lipotoxicity, in which excessive fatty acid accumulation leads to lipid intermediates and cardiomyocyte apoptosis (25, 26); (ii) inflammation, with increased pro-inflammatory macrophages and cytokines alongside reduced anti-inflammatory adipokines (26, 27, 30); (iii) oxidative stress, driven by local hypoxia and reactive oxygen species activating innate immune responses (25, 28); and (iv) loss of browning (whitening), with downregulated UCP-1 expression and a promoted pro-inflammatory phenotype (28, 30).

Research by Gruzeva et al. shows an imbalance in pro-inflammatory/anti-inflammatory cytokine expression in EAT of obese patients: elevated levels of pro-inflammatory factors (such as IL-6, IL-17, TNF-α, and leptin), while secretion of the anti-inflammatory factor adiponectin is reduced. This imbalance exacerbates local inflammation and oxidative stress, thereby promoting inflammatory responses and fibrotic remodeling in the coronary arteries and myocardium, leading to the development of cardiovascular diseases (31). In individuals with obesity, metabolic syndrome, or diabetes, EAT becomes a harmful factor promoting the development of cardiovascular disease (26). Studies have also found that compared to the control group and pre-heart failure, patients with heart failure with preserved ejection fraction (HFpEF) have significantly increased EAT volume (*P* < 0.001), and these patients with elevated EAT often present with characteristics such as diabetes, ischemia, and inflammation (32). In Lipson et al.'s research report, it is stated that the EAT volume of SLE patients increased, higher than that of the control group, and the coronary artery calcification score is positively correlated with the EAT volume (33). This study also yielded similar results, with the case group showing higher PLAX EAT, PMPSAX EAT, P4C EAT, A4C EAT, and mean EAT compared with the control group. There was a statistically significant difference in EAT changes between the two groups (*P* < 0.05).

What is more important is that this study found a significant negative correlation between EAT and left ventricular longitudinal strain functional parameters in patients with SLE, with EAT being an independent influencing factor of left ventricular longitudinal strain function. Previous studies have reported a negative correlation between EAT and left ventricular systolic dysfunction, with an increase in EAT thickness associated with subclinical systolic dysfunction (34). Increased EAT thickness is associated with reduced left ventricular end-diastolic diameter, increased left ventricular wall thickness, and decreased GLS, and for each one standard deviation increase in EAT thickness, the risk of heart failure significantly increases (*P* < 0.001) (35).

### The relationship between regional strain analysis and corresponding coronary blood supply and its association with EAT

Previous studies have shown that EAT volume is increased in SLE patients (33) while GLS is decreased (22); however, as a global parameter, GLS is unable to reveal regional heterogeneity. This study further refines the analysis dimension from overall GLS to segmental strain in each coronary artery supply region, dividing the left ventricle into 18 segments and grouping them according to coronary artery supply regions.

We found that compared with the control group, LAD, RCA, and LCX values were all lower in the case group (*P* < 0.05), and each of these parameters showed a significant negative correlation with EAT thickness. This indicates that the decrease in myocardial strain in SLE patients is across all coronary blood supply regions, presenting as diffuse and uniform, rather than selectively limited to myocardial segments dominated by a single coronary artery, which distinguishes it from traditional coronary heart disease. This finding supports the hypothesis that EAT directly injures adjacent myocardium via a paracrine mechanism, from the perspective of spatial association. This unique anatomical location enables EAT to secrete abundant bioactive molecules and directly influence adjacent myocardium and coronary arteries via paracrine mechanisms (25, 27). Its pro-inflammatory mechanism can directly affect the contractility of the underlying myocardium, thereby playing a role in the development of left ventricular dysfunction.

### EAT thickening and pathological mechanisms associated with SLE inflammation

The thickening of EAT in SLE patients is not simply fat accumulation but rather a localized manifestation of systemic chronic inflammation within the heart-specific fat depot. Against the background of persistent immune activation and elevated pro-inflammatory cytokine levels in SLE, EAT transforms from a protective tissue into a pro-inflammatory tissue, characterized by significant increases in thickness and volume, and correlates with disease activity (4, 27, 30). This abnormal deposition not only reduces the pericardial cavity volume and restricts cardiac motion, but more importantly, mediates cardiac dysfunction through multiple mechanisms.

First, EAT thickening is accompanied by immune cell infiltration and phenotypic transformation: Macrophages within EAT shift from anti-inflammatory M2 polarization to pro-inflammatory M1 polarization; the imbalance in the M1/M2 ratio correlates with the severity of coronary artery disease (36). Meanwhile, infiltration of mast cells, CD8+ T lymphocytes, and Th17 cells increases, further exacerbating the local inflammatory response (5, 26, 28, 29). Second, hypertrophied EAT secretes large amounts of pro-inflammatory cytokines (such as IL-1β, IL-6, and TNF-α), which induce cardiomyocyte apoptosis and dysfunction via paracrine signaling (13, 37, 38). IL-1β increases the negative inotropic effect by affecting Ca^2^⁺ regulatory proteins (38), while TNF-α promotes myocardial fibrosis (39). In addition, EAT thickening is also accompanied by adipokine imbalance. Decreased adiponectin expression weakens its inhibitory effect on the NF-κB pathway, while increased expression of leptin and resistin promotes endothelial dysfunction and oxidative stress (4, 5, 28, 30, 36).

At the levels of oxidative stress and mitochondrial function, EAT thickening mutually promotes increased reactive oxygen species (ROS) production, downregulation of antioxidant enzymes, and mitochondrial dysfunction, leading to disordered myocardial energy metabolism and apoptosis (13–15, 29, 37, 38). Meanwhile, activation of the AGEs-RAGE signaling pathway in EAT further amplifies inflammatory and oxidative stress responses (4, 27, 30). EAT thickening not only contributes to myocardial fibrosis and electrophysiological remodeling but also directly participates in these processes: The secreted TGF-β and HIF-1 promote fibroblast activation and collagen deposition (13, 15, 37). An imbalance of MMP-2, MMP-9, and TIMPs mediates extracellular matrix remodeling (14, 38, 39). Involvement of autonomic ganglia induces atrial electrophysiological remodeling, increasing the risk of atrial fibrillation (25, 26, 29, 40, 41). Ultimately, via pericardial constraint, impaired ventricular filling, and myocardial dysfunction, EAT thickening contributes to heart failure with preserved ejection fraction and ventricular systolic–diastolic dysfunction (4, 27, 28, 30, 36).

In summary, SLE-driven systemic inflammation promotes EAT thickening and functional dysregulation through multiple pathways, including immune cell infiltration, activation of cytokine networks, adipokine imbalance, oxidative stress, and mitochondrial dysfunction, thereby establishing a vicious cycle of “systemic inflammation–local EAT activation–cardiac dysfunction.”

It should be emphasized that not all inflammatory conditions lead to the same pattern of epicardial adipose tissue (EAT) remodeling. In systemic lupus erythematosus (SLE), sterile inflammation driven by autoimmune mechanisms promotes pathological EAT remodeling. In contrast, infectious inflammation—such as that induced by Mycobacterium tuberculosis—activates Toll-like receptor 2 (TLR2) and TLR4 signaling pathways through pathogen-associated molecular patterns (PAMPs), triggering a Th1-predominant immune response characterized by the production of interferon (IFN)-γ*,* tumor necrosis factor (TNF)-α, and granuloma formation (42). Although both conditions may present with local inflammatory infiltration and EAT thickening, they are fundamentally distinct: SLE is marked by self-reactive T-cell-mediated persistent tissue injury, whereas infectious inflammation prioritizes pathogen clearance and host defense. Therefore, SLE-associated EAT thickening has a unique immunopathological basis, and findings from SLE studies should not be directly extrapolated to other inflammatory diseases.

These findings indicate that there is a direct gradient association between EAT thickness and the severity of cardiac function impairment. This provides preliminary clinical evidence that EAT directly participates in or exacerbates changes in cardiac function, rather than merely being a comorbid condition. Moreover, this injury is diffuse and lacks the selective pattern typical of coronary artery distribution. The above-mentioned research findings further establish the value of EAT as an imaging biomarker for cardiovascular risk assessment in SLE patients. Combined assessment with strain parameters such as GLS helps to identify SLE patients with subclinical myocardial involvement at an earlier stage and provides a basis for clinical intervention.

### Limitations

This study has several limitations. This study cannot infer causality; it is possible that EAT thickness leads to decreased cardiac function in SLE patients, or that decreased cardiac function and metabolic changes result in increased EAT thickness, or that both share a common cause. Although mechanistic hypotheses—including immune cell infiltration and adipokine imbalance—have been proposed, the levels of inflammatory cytokines in serum or local adipose tissue were not measured. Future studies integrating animal experiments or histopathological examinations are warranted to further elucidate the underlying molecular mechanisms. Although GLS is superior to LVEF in assessing left ventricular systolic function, the current differences in speckle-tracking algorithms among various ultrasound equipment manufacturers mean that GLS measurement has not yet been fully standardized. Future multicenter, multi-device validation studies are therefore warranted.

The sample size of this study was relatively small, and it also did not include more inflammatory markers. It is recommended to carry out prospective cohort studies, increase the sample size, measure the changes in EAT thickness, and conduct long-term follow-up of heart function changes to clarify the sequence of events and the causal relationship. Combine with more refined clinical research, measure inflammatory factors, and explore the mechanisms in depth.

This study included only adult patients with SLE; therefore, the applicability of our findings to pediatric SLE requires further validation. Notably, cardiac involvement in pediatric SLE predominantly manifests as myocarditis, whereas myocardial infarction is more characteristic in adults (43). Pediatric SLE is associated with a more aggressive disease trajectory, increased cardiovascular risk, and earlier onset of organ damage (44). Previous studies have shown that subclinical left ventricular systolic and diastolic dysfunction may already exist in children with SLE (45). Thus, future studies are warranted to independently validate the association between epicardial adipose tissue and left ventricular function, specifically in pediatric SLE populations, to determine the generalizability of our conclusions.

## Conclusions

This study utilized 2D-STE to conduct an in-depth analysis by grouping the left ventricular 18-segment longitudinal strain of SLE patients according to the three coronary artery supply territories—LAD, RCA, and LCX—and systematically explored the relationship between EAT thickness and myocardial strain in various regions of SLE patients. The main conclusions are as follows: (1) SLE patients exhibited a significant increase in EAT thickness, along with significant reductions in GLS, AP2C LS, AP3C LS, and AP4C LS—changes that occurred earlier than alterations in conventional cardiac functional parameters such as LVEF. This indicates changes in EAT and early myocardial injury in SLE patients. Two-dimensional STE is beneficial for early assessment of cardiac functional changes. (2) In SLE patients, EAT thickness is significantly negatively correlated with AP2C LS, AP3C LS, AP4C LS, and GLS, and EAT is an important influencing factor of left ventricular longitudinal strain function. There appears to be a gradient correlation between EAT and the degree of cardiac functional impairment, suggesting a close association rather than a direct causal gradient. (3) In SLE patients, the left ventricular segmental longitudinal strain corresponding to the perfusion territories of the LAD, RCA, and LCX was significantly lower than in healthy controls, and EAT showed a negative correlation with it among patients. In patients with SLE, left ventricular segmental longitudinal strain values for the myocardial perfusion territories supplied by the LAD, RCA, and LCX were significantly lower than those in healthy controls; among these patients, EAT showed a significant negative correlation with segmental longitudinal strain.

In light of the cross-sectional design of this study, the findings should be interpreted as demonstrating significant correlations rather than causal relationships. Several core findings of this study mutually correlate with each other, collectively revealing the potential role of EAT in the early impairment of left ventricular systolic function in SLE patients. EAT may serve as an imaging marker of cardiovascular risk in SLE patients and may be closely related to the pathological process of impaired myocardial contractile function. The echocardiographic strategy combining EAT assessment with left ventricular longitudinal strain analysis helps to identify subclinical myocardial damage earlier and more comprehensively in SLE patients, optimizing individualized cardiovascular risk management. Further prospective studies are warranted to clarify the underlying mechanisms and temporal sequence of these associations.

## Data Availability

The raw data supporting the conclusions of this article will be made available by the authors, without undue reservation.
